# Factors Impacting the Use or Rejection of Hearing Aids—A Systematic Review and Meta-Analysis

**DOI:** 10.3390/jcm12124030

**Published:** 2023-06-13

**Authors:** Susana Marcos-Alonso, Cristina Nicole Almeida-Ayerve, Chiara Monopoli-Roca, Guillermo Salib Coronel-Touma, Sofía Pacheco-López, Paula Peña-Navarro, José Manuel Serradilla-López, Hortensia Sánchez-Gómez, José Luis Pardal-Refoyo, Ángel Batuecas-Caletrío

**Affiliations:** 1 Department of Otorhinolaryngology—Head & Neck Surgery, Hospital Universitario de Salamanca, 37007 Salamanca, Spain; 2 Biomedical Research Institute of Salamanca (IBSAL), Faculty of Medicine, The University of Salamanca, 37007 Salamanca, Spain

**Keywords:** hearing aid adherence, hearing aid rejection, sensorineural hearing loss, hearing aids

## Abstract

Purpose: To examine the prevalence of adherence to hearing aids and determine their rejection causes. Methods: This study was conducted according to the Preferred Reporting terms for Systematic Reviews and Meta-Analyses (PRISMA) guidelines. We performed an electronic search using PubMed, BVS, and Embase. Results: 21 studies that met the inclusion criteria were selected. They analyzed a total of 12,696 individuals. We observed that the most common causes for positive adherence to hearing aid use included having a higher degree of hearing loss, patients being aware of their condition, and requiring the device in their daily life. The most common causes for rejection were the lack of perceived benefits or discomfort with the use of the device. The results from the meta-analysis show a prevalence of patients who used their hearing aid of 0.623 (95% CI 0.531, 0.714). Both groups are highly heterogeneous (I2 = 99.31% in each group, *p* < 0.05). Conclusions: A significant proportion of patients (38%) do not use their hearing aid devices. Homogeneous multicenter studies using the same methodology are needed to analyze the causes of rejection of hearing aids.

## 1. Introduction

Currently, the rise in life expectancy has been accompanied by the unavoidable development of age-related concomitant diseases and morbidity, including hearing loss, which has a significant impact on life quality [1] and is one of the most common chronic conditions in adults over 65 years old [2], combined with the functional deficiency caused by biological and functional changes [3].

Hearing loss suggests worsening cognitive deficit and mental processing, which could cause isolation and psychopathological problems, making integration into society more difficult [3,4]. Presbycusis is characterized by sensorineural hearing loss of unknown origin related to the aging of ganglions and ciliated cells. One of the treatments for sensorineural hearing loss is the use of hearing prostheses, and one of the most common categories within this group is hearing aids, which can significantly increase the quality of life of these patients [5]. However, not all patients finally use their hearing aids [6].

There has been a constant 26% increase in years lived with disability for age-related and other types of hearing loss, which explains the rise in disability-adjusted life-years. In 2019, an estimated 1.57 billion people had hearing loss, accounting for one in five people. Of these, approximately 403 million people had hearing loss that was moderate or higher in severity after adjusting for hearing aid use, and 430 million without adjustment. Of all people with a hearing impairment, 62.1% were older than 50 years. By 2050, a projected 2.45 billion people will have hearing loss, a 56.1% increase from 2019, despite stable age-standardized prevalence [7,8].

There are different articles in the literature that attempt to analyze the causes of why these patients with sensorineural hearing loss, who are hearing aid candidates, use or reject hearing aids. Despite significant developments and improvements in hearing aids over the last few years [9], some mentioned factors are related to the device mold, lack of maintenance, noise complaints, unmet expectations, or lack of a subjective improvement in the patient’s problem [10] and data show that between 3% and 16% of the patients ultimately return their devices [11].

Dillon et al. (2020) showed significant variability in estimates of the non-use of hearing aids (mainly in studies conducted in developed countries). It is possible that, in addition to the variability between studies from different countries with different socio-demographic contexts, there is also variability within the same country. For instance, in the UK, the study by Aazh et al. (2015) estimated non-use at 10%, while Dillon et al. (2020) observed a prevalence of non-use at 18% [12,13].

Knudsen et al. performed an extensive search of the literature covering studies addressing help-seeking, hearing aid uptake, use, and satisfaction with hearing aids yielded 39 empirical studies published between January 1980 and January 2009. A total of 31 factors were examined in these studies. These were personal factors (e.g., source of motivation, expectation, attitude, hearing sensitivity), demographic factors (e.g., age, gender), or external factors (e.g., cost, counseling). Whereas they found mixed results for most factors, they found that self-reported activity limitation as measured before treatment correlated positively with all four outcomes throughout all stages (prefitting, fitting, postfitting). This is a noteworthy finding demonstrating that self-reported auditory difficulty is highly important in aural rehabilitation, possibly more important than objective hearing sensitivity, which showed less consistent effects on outcomes. This result underlines the need to carefully consider what outcome (help-seeking, uptake, use, or satisfaction) is the prime interest when designing rehabilitation programs. They conclude that whereas there is a large body of literature available on factors possibly influencing the different stages in the process toward help-seeking, obtaining a hearing aid, using it, and becoming satisfied with it, there are many issues that have not yet been investigated in controlled studies [14].

Ng et al. reviewed a total of twenty-two articles. The identified four audiological determinants (the severity of hearing loss, the type of hearing aids, background noise acceptance, and insertion gain) and seven non-audiological determinants (self-perceived hearing problems, expectation, demographics, group consultation, support from significant others, self-perceived benefit, and satisfaction) as factors affecting the adoption and use of hearing aids. They concluded that there was a need to explore the influence of significant others, health professionals, and user demographics on hearing rehabilitation for future research [15].

Our study aims to address the gaps identified in the systematic reviews mentioned above. We present a systematic review that analyzes the determining factors behind patients’ decisions to either reject or use their hearing aids.

## 2. Methods

### 2.1. Search Strategy

This study was conducted according to the Preferred Reporting terms for Systematic Reviews and Meta-Analyses (PRISMA) guidelines [16]. Following the NICE recommendations, we formulated the research question as follows: what factors influence the decision to accept or reject hearing aid fitting in adult patients with deafness? [17].

In our study, we conducted a comparison between group A (consisting of individuals who adhered to the use of hearing aids) and group B (individuals who rejected the use of hearing aids). In group A, we included individuals who used their hearing aids, regardless of the duration of the use. In group B, we included individuals who either kept the device but did not use it or returned it.

A comprehensive search strategy was conducted using the PubMed, Embase, and BVS (Biblioteca Virtual de Salud=Virtual Health Library, VHL) databases. We used a combination of medical subject headings and keywords to cover the following concepts: hearing aid, older adults, elderly, patient compliance, use, adherence, and acceptance. The search strategy was adapted to each concept to ensure comprehensive coverage of the relevant literature. The most recent search was conducted on 7th February 2022. The Boolean operators used are shown in Table 1.

### 2.2. Selection Criteria

We included studies that described the causes of the use or rejection of hearing aids based on the following criteria. For all eligible articles, data extraction was performed, and basic information was extracted.

Inclusion criteria: publications from the last 25 years on adherence to hearing aid use in adult patients (over 18 years old), including the total number of patients with hearing loss, hearing aid candidacy, use or rejection of hearing aid, and its associated factors. The initial selection of the articles was based on the title and abstract of the articles, followed by a full-text assessment. The articles considered were those written in English, French, or Spanish. Articles had to include the following variables: age and gender of the participants, causes for use or rejection of the hearing aids, and total number of patients who used and who rejected the devices.

All studies that did not meet the inclusion criteria were excluded.

The search and article selection processes were independently conducted by two authors using the Rayyan tool [18].

### 2.3. Qualitative and Quantitative Data Synthesis and Statistical Analysis

A descriptive analysis was conducted to examine the factors related to the use or rejection of hearing aids. This analysis included continuous variables (number of patients who used or rejected the hearing aid, mean age), Student’s T-test for two independent samples was employed, and a meta-analysis of risk difference was conducted using a random-effect model to compare the prevalence of use or rejection of the hearing aid. The homogeneity and publication bias of the study were assessed. The statistical software JAMOVI was used along with the statistical packages R and MAJOR (The Jamovi project. Version 2.2, 2021). The estimation of proportions for each study employed the restricted maximum likelihood as the model estimator, and the effect size model measure used the raw proportion. The restricted maximum likelihood method was also used. The methodological quality of each study was assessed using the ROBINS-I scale [19].

## 3. Results

**Figure 1:**
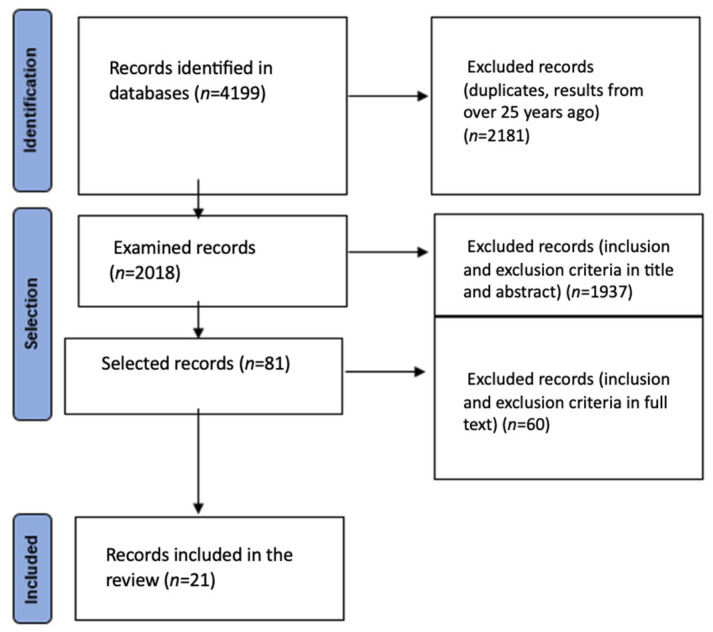
The PRISMA flow diagram.

Out of the initial 4199 articles identified through the search, 81 studies remained after screening titles and abstracts (refer to Figure 1). After conducting a full-text review, 21 studies that met the inclusion criteria were selected for analysis. These studies collectively analyzed data from 12,696 patients with an average age of 72.2 years (95% CI: 69.6, 74.7 years). The age range of the participants varied from 18 to 101 years (see Table 2).

In each article, various factors were associated with the rejection or use of hearing aids. Since these factors varied across the articles, we tried to homogenize the variables by grouping them. This allowed for a more consistent and comparable analysis of the factors influencing the decision to reject or use hearing aids across the selected studies.

For the use of the hearing aids, we found factors that were grouped into the following categories: (1) higher degree of hearing loss or bilateral hearing loss. The classification of the hearing loss degree varied across the screened articles; (2) greater awareness of their condition; (3) greater satisfaction with the device, ease of use of the device, or having a simpler or more modern device; (4) higher economic status, higher income, higher level of education, having a university degree (the definition of high economic level may vary across the examined articles); (5) social issues (working age, daily activity, difficulties communicating in daily life, social support); (6) emotional causes, having a positive attitude; (7) greater experience using the device, using the device for more hours per day; (8) being Caucasian; (9) recent diagnosis, recent audiometry evaluation, regular medical monitoring; (10) older age; (11) being male; and (12) other comorbidities.

Similarly, the causes for rejection were grouped as follows: (1) lack of awareness of their condition. This category includes factors related to individuals not being fully aware of their hearing loss condition or its impact on their daily life; (2) low or no perceived benefits from hearing aid use; (3) inability to understand others; (4) finding the device uncomfortable or difficult to use; (5) social stigma and other social causes; (6) lack of sufficient income. This factor relates to individuals not having enough financial resources to afford hearing aids or related costs; (7) lack of social or family support; and (8) older age.

Based on the analysis of the selected studies, the most common causes for positive adherence to hearing aid use were identified as follows: (1) a higher degree of hearing loss: individuals with a more severe degree of hearing loss were more likely to adhere to the use of hearing aids; (2) awareness of their condition and daily necessity: individuals who were aware of their hearing loss condition and recognized the need for the device in their daily life were more likely to adhere to using hearing aids; (3) adequate income: having a higher income or sufficient financial resources was associated with positive adherence to hearing aid use; (4) greater experience using the device: individuals who had more experience using hearing aids and were familiar with their operation and benefits were more likely to adhere to their use. On the other hand, the most common causes for rejection of hearing aids were: (1) lack of perceived benefits: individuals who did not perceive significant benefits or improvements in their hearing or communication abilities with the use of hearing aids were more likely to reject them; (2) discomfort with the device: some individuals found the device uncomfortable to wear or experienced difficulties in using it, leading to rejection. Additionally, being older was identified as a factor that could both contribute to positive adherence to hearing aid use and rejection. The reasons for this dual association may vary and could be influenced by factors such as individual preferences, attitudes, or specific needs related to age (Table 3).

**Figure 2:**
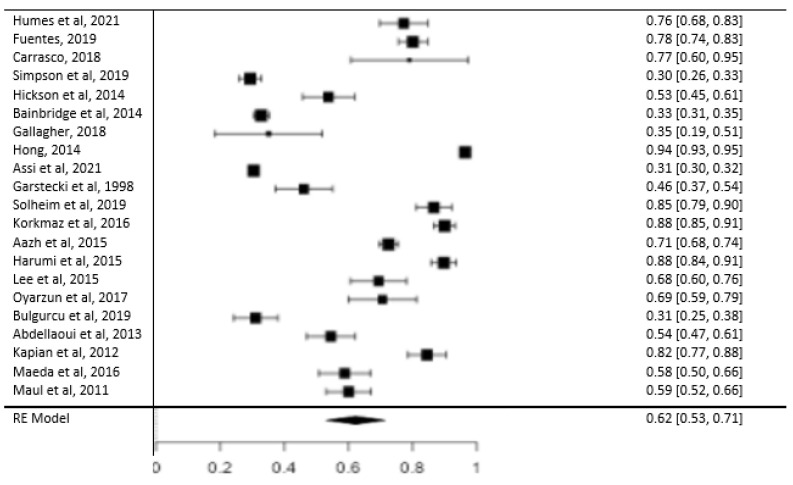
Forest plot showing prevalence in group A [10,12,20,21, 22,23,24,25,26,27,28,29,30,31,32,33,34,35,36,37,38].

**Figure 3:**
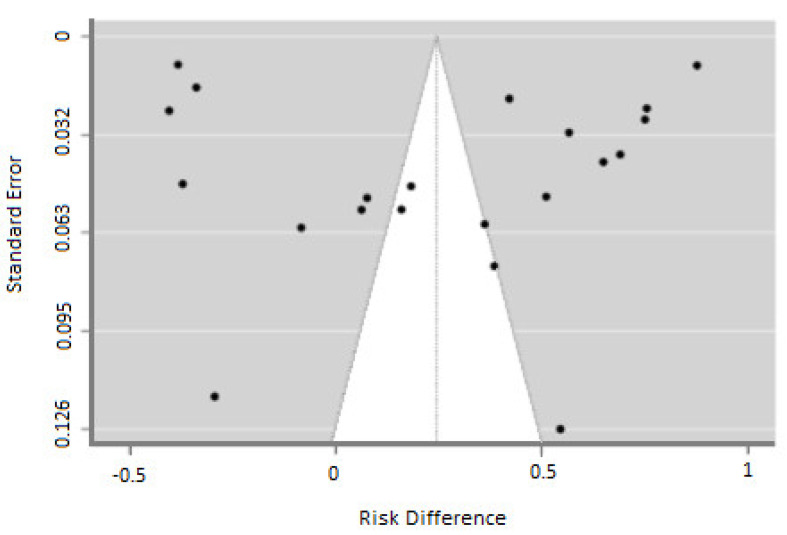
Funnel plot showing publication bias.

**Figure 4:**
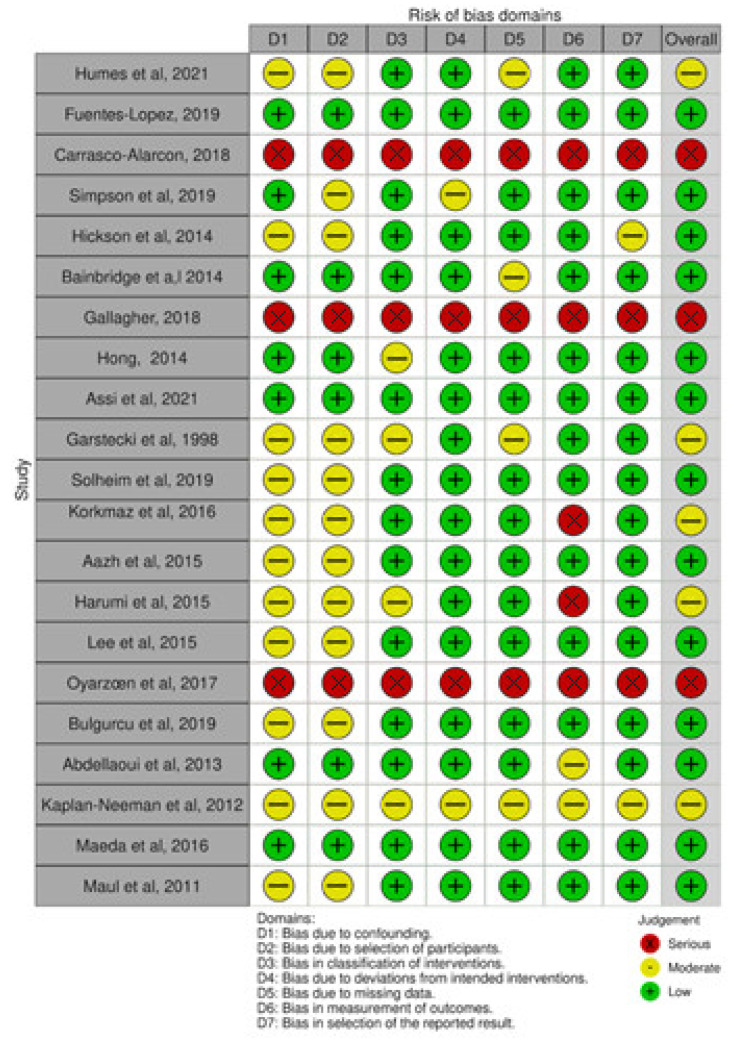

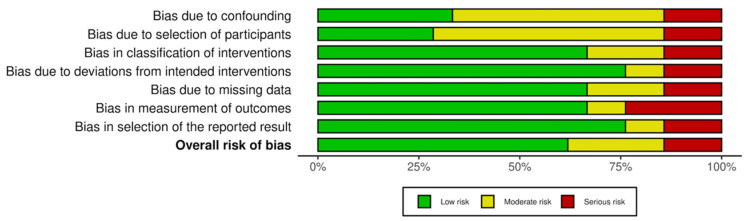
Assessment of the methodological quality of each study applying the ROBINS-I scale [10,12,20,21, 22,23,24,25,26,27,28,29,30,31,32,33,34,35,36,37,38].

The meta-analysis results indicate a prevalence of patients who used the hearing aid (group A) of 0.623, with a 95% confidence interval (CI) ranging from 0.531 to 0.714. The analysis also reveals high heterogeneity within both group A and group B, as indicated by an I2 value of 99.31, a Tau value of 0.211, and a significant Q value of 6421.241 (*p* < 0.05) (Figure 2 and Figure 3). The analyses show that there is a publication bias in both groups (Figure 2 and Figure 3). Figure 4 presents the results of the ROBINS-I scale, which assesses the methodological quality of each included study. The scale provides an evaluation of the risk of bias in the included studies, helping to determine the overall quality of evidence and potential sources of bias in the results.

## 4. Discussion

The literature reveals various factors that contribute to the use or rejection of hearing aids, encompassing demographic, clinical, and device-related aspects. The rate of rejection typically falls within the range of 20% to 30%. However, if we consider patients who only use their hearing aids for a few hours a day, the rejection rate can increase to 40% [12]. Adherence tends to be lower in patients over 80 years old who use bilateral devices [12]. A long time of use of the device is associated with more daily use [26,30,31,32,35]. Studies have shown that a longer duration of device use is associated with increased daily usage [26,30,31,32,35]. Maul et al. support these findings and assert that approximately half of the patients prescribed hearing aids do not utilize them. They emphasize that addressing presbycusis remains a challenging issue that requires a comprehensive and interdisciplinary approach to ensure optimal management for individuals and achieve sufficient coverage and performance on a population level [10].

Regarding device-related factors, Fuentes López et al. found that 21.7% of patients discontinued using their hearing aids, with the majority ceasing usage within the first six months. The factors associated with increased usage were socioeconomic status, perceived degree of hearing loss, and satisfaction with the device [21]. On the other hand, Carrasco Alarcón et al. observed a 75% adherence rate, with problems related to the device mold being the main cause for rejection. They concluded that better adjustments and calibration could improve adherence [22]. Humes et al. emphasized the importance of manual skills to ensure better adherence, enabling patients to use the device independently without relying on others [20]. Hickson et al. highlighted the positive impact of support from others in promoting the use of hearing aids. They also emphasized the need for specific questionnaires to assess the reasons for using hearing aids [24].

The literature consistently highlights that a higher degree of hearing loss and greater awareness of one’s condition and perceived hearing loss are important factors that promote the use of hearing aids. Patients with moderate hearing loss are reported to use their hearing aids more frequently than patients with other degrees of hearing loss [39].

Lee et al. identified individuals with daily professional and/or social activities for whom hearing loss significantly impacts their daily life as predictive factors for hearing aid use. These individuals have a greater awareness of their condition and recognize the necessity of using hearing aids [33].

Simpson et al. found that it takes patients approximately nine years from the time of prescription to decide to use hearing aids. They also identified perceived needs and the social handicap associated with hearing loss as motivating factors for increased hearing aid use [23].

A higher degree of hearing loss has been consistently associated with seeking help and a greater likelihood of using hearing aid devices [14,40]. McCornack et al. found in their 2013 study that one of the primary reasons for the non-use of hearing aids is the patients’ lack of awareness of their hearing loss. This lack of awareness can lead to a delay in seeking treatment and accepting the use of hearing aids [41]. Maeda et al. emphasized the significance of emotional factors in the use of hearing aids. Emotional factors, such as self-perception, self-esteem, and the impact of hearing loss on social interactions, can influence an individual’s motivation and commitment to using hearing aid devices [38].

While most studies do not report significant differences between ethnic groups in terms of hearing aid use, there is some evidence suggesting that Black patients may use hearing aid devices less frequently compared to White patients [25,42,43]. These findings indicate a potential disparity in access to and utilization of hearing aids among different racial and ethnic groups. Regarding sex differences, it has been observed that a small percentage of patients, approximately 6%, return their hearing aids within the first three months of use, and this trend is more pronounced among women [27]. Regarding sex differences, the observation that a higher percentage of women return their hearing aids within the first three months of use suggests a potential disparity in their experience and satisfaction with the devices compared to men. The specific reasons for this sex difference in return rates, such as comfort, fit, or personal preferences, need further investigation to better understand and address the underlying factors contributing to this disparity.

Regarding age, there seems to be variation in the factors influencing the use of hearing aids among different age groups. Younger patients may face economic challenges that hinder their ability to use hearing aids effectively, while older patients may encounter difficulties handling the devices. However, it is worth noting that different studies have provided conflicting findings regarding the relationship between age and hearing aid use. Some studies suggest that being younger is associated with a higher likelihood of using the device, driven by the need and willingness to rehabilitate [44], while others report that use increases proportionally with age [34,40]. These inconsistencies highlight the need for further research to gain a clearer understanding of the impact of age on hearing aid use and the underlying factors influencing it.

Income and economic status have been identified as significant factors related to the rejection of hearing aid devices. Bainbridge et al. conducted a study on patients over 70 years old and found that the main reason for rejecting the device in their sample was the lack of sufficient income. They emphasized the importance of the public health system assisting these patients [25]. Similarly, Lama et al. also highlighted the need for support and assistance for individuals with limited financial resources to ensure access to hearing aids [28]. These findings underscore the importance of addressing economic barriers and ensuring affordable options for individuals who may otherwise reject or be unable to obtain hearing aids due to financial constraints.

The role of doctors and healthcare professionals in promoting adherence to hearing aid use is crucial. Studies have shown that better adherence is associated with more frequent medical consultations and closer monitoring by healthcare professionals. Providing detailed information and regular monitoring increases patients’ motivation to seek information, try the devices, and ultimately use them [26,28,30,31,32,35]. Abdellaoui et al. found that one-third of patients with age-related hearing loss did not complete the follow-up of their hearing aid prescription with ENT services, citing reasons such as economic constraints or lack of motivation. Therefore, when providing recommendations, doctors must consider patients’ motivation, immediate environment, awareness of their disability, and income, as these factors can influence their choice of device and subsequent purchases [36]. ENT doctors play a significant role as they prescribe the use of hearing aids and serve as the initial assessors. While their role may be limited in the follow-up period, their involvement is fundamental in the decision-making process of acquiring a hearing aid device. Kaplan-Neeman et al. emphasize the importance of doctors informing their patients that greater satisfaction with the device can be achieved by using it for more hours each day [37]. Overall, healthcare professionals, especially ENT doctors, play a critical role in educating and supporting patients throughout the process of adopting and using hearing aids. Regular consultations, effective communication, and ongoing monitoring can contribute to increased adherence and improved patient outcomes.

### Limitations of the Study

Including people over 18 years old may introduce significant heterogeneity in the prevalence estimates due to the wide age range and potential differences in factors related to hearing aid use. While the original intention was to focus solely on the elderly population, the decision to include individuals over 18 years old was made due to the larger number of articles available for analysis [45]. Future research focusing specifically on the elderly population may provide more targeted and applicable insights for this age group.

Including studies from the past 25 years may introduce additional heterogeneity due to the significant changes in hearing aid technology during this period. As previously mentioned, the technology of hearing aid devices is a variable associated with their use, and advancements in technology can impact the prevalence and factors related to their use [15]. Studies conducted earlier within the 25-year time frame may not accurately represent the current landscape of hearing aid use and associated variables. It is important to consider the potential impact of this time cut-off on the generalizability of the study’s findings to the current population using hearing aids.

It is important to acknowledge that estimating a grouped prevalence with high heterogeneity may not provide specific and accurate information about any particular population subgroup. The estimate of 63.2% adherence to hearing aid use may represent an average prevalence based on the included studies, but the high heterogeneity suggests significant variability in the prevalence rates across different populations. Migliavaca et al. (2022) [46] highlight that in meta-analyses of prevalence, the summary estimate represents an average prevalence based on the included studies. This average estimate can be informative if there is minimal heterogeneity among the different prevalence rates. However, in cases where there is substantial heterogeneity, the summary estimate may not accurately reflect the prevalence in any specific subgroup [19]. Considering the high heterogeneity observed in the analysis, it is important to interpret the estimate with caution and acknowledge the potential limitations in generalizing the findings to specific population groups. Future research with more focused and homogeneous population samples could provide more precise prevalence estimates that apply to specific subgroups.

It is also important to acknowledge that the variability in the information collected and reported by different studies can limit the comparability of results.

There is also a high risk of bias, as measured by the ROBINS tool. The prevalence estimates presented in this review are based on the dichotomization between “people who use their hearing aids any amount of time” and “people who kept the device but did not use it at all or returned them”. However, the primary studies included in this review did not consistently use this dichotomization when studying variables associated with adherence. For example, Oyarzún et al. (2017) [34] used the IOI-HA questionnaire score as a continuous variable, while Aazh et al. (2015) [12] dichotomized it based on hours of use per day. Additionally, Hickson et al. (2014) [24] used a criterion composed of questions on adherence and perceived benefit, which differed from the approach used in this manuscript. Therefore, the variables associated with the prevalence estimates used may not align with the dichotomization used in this study and may not be valid.

The heterogeneity among the studies may also be attributed to the use of different questionnaires by different authors. For instance, Aazh et al. (2014) [12] used the IOI-HA questionnaire along with self-constructed questions, while Fuentes-López et al. (2019) [21] used the Bertoli et al. (2009) [47] questionnaire. Carrasco-Alarcón et al. (2018) [22] employed a self-constructed 12-question survey without prior validation. In the case of Simpson et al. (2019) [33], the estimates were based on a comparison between those who never adopted hearing aids and those who adopted them during the study, which introduces another source of heterogeneity and deviates from the authors’ definition of adherence.

Prospective studies with a homogeneous methodology focusing on the population over 65 years old are needed to establish the causes, and additional data on age, sex, income, and education are necessary to define specific profiles for each group.

## 5. Conclusions

- A significant proportion of patients (38%) do not use their hearing aid devices.
- Being aware of the need to use hearing aids and having a higher degree of hearing loss are important factors that promote their use.
- Homogeneous multicenter studies using the same methodology are needed to analyze the causes of rejection of hearing aids.
- Due to the heterogeneity and bias in the studies, it is not possible to objectively establish the causes for the use of hearing aids, and the quality of the evidence is low. There is heterogeneity among the studies included in this analysis, including non-randomized series, variation in the populations studied, different study designs, and diverse data collection methods. The wide age range and the inclusion of studies conducted over a long period, which encompasses changes in technology and social habits, further contribute to the heterogeneity. This increased heterogeneity suggests that the results may not apply to the overall population. Therefore, valid conclusions cannot be drawn regarding the characteristics and profiles of patients in each group.

## Figures and Tables

**Table 1:** Boolean operators. (BVS=Biblioteca Virtual de Salud, Virtual Health Library).

Database	Boolean Operators
EMBASE	(‘hearing aid’/exp OR ‘hearing aid’ OR ((‘hearing’/exp OR hearing) AND (‘aid’/exp OR aid))) AND (‘adaptation’/exp OR adaptation) (‘hearing aid’/exp OR ‘hearing aid’ OR ((‘hearing’/exp OR hearing) AND (‘aid’/exp OR aid))) AND (‘patient compliance’/exp OR ‘patient compliance’) ((‘hearing aid’/exp OR ‘hearing aid’ OR ((‘hearing’/exp OR hearing) AND (‘aid’/exp OR aid))) AND (‘patient compliance’/exp OR ‘patient compliance’) OR ‘acceptance’/exp OR acceptance) AND (‘older adults’/exp OR ‘older adults’) (‘hearing aid’/exp OR ‘hearing aid’ OR ((‘hearing’/exp OR hearing) AND (‘aid’/exp OR aid))) AND (‘acceptance’/exp OR acceptance) AND (‘older adults’/exp OR ‘older adults’) (‘aged’/exp OR ‘aged’ OR ‘aged patient’ OR ‘aged people’ OR ‘aged person’ OR ‘aged subject’ OR ‘elderly’ OR ‘elderly patient’ OR ‘elderly people’ OR ‘elderly person’ OR ‘elderly subject’ OR ‘senior citizen’ OR ‘senium’) AND (‘hearing aid’/exp OR ‘aid, hearing’ OR ‘auditory appliance’ OR ‘auditory prosthesis’ OR ‘fitting, hearing aid’ OR ‘hearing aid’ OR ‘hearing aid device’ OR ‘hearing aid fitting’ OR ‘hearing aid, device’ OR ‘hearing aids’ OR ‘hearing apparatus’ OR ‘hearing device’ OR ‘listening aid’ OR ‘listening aids’) AND (‘patient compliance’/exp OR ‘adherence to therapy’ OR ‘adherence to treatment’ OR ‘compliance to therapy’ OR ‘compliance to treatment’ OR ‘patient adherence’ OR ‘patient compliance’ OR ‘patients’ adherence’ OR ‘therapy adherence’ OR ‘therapy compliance’ OR ‘treatment adherence’ OR ‘treatment adherence and compliance’ OR ‘treatment compliance’)
PUBMED	hearing aid AND older adults AND patient compliance hearing aid AND elderly AND use hearing aid and elderly and use hearing aid and elderly and adherence hearing aid and elderly and acceptance hearing aid and elderly and acceptance hearing aid and use and covid hearing aid AND elderly AND (use OR adherence OR acceptance OR Patient compliance)
BVS	hearing aid and elderly and patient compliance hearing aid and elderly and acceptance hearing aid and elderly and adherence

**Table 2:** The 21 studies that met the inclusion criteria showed a total of 12,696 patients.

Author, Year		Total No.	Adherence (n)	Rejection n (Rate)	AVG. AGE	Min. Age	Max. Age	Avg. Age Use	Avg. Age Rejection
Humes et al., 2021 [20]	Retrospective	139	105	34 (24%)	73.7	60	89	74.7	72.7
Fuentes-López, 2019 [21]	Prospective	355	278	77 (22%)	74.9	65	85		
Carrasco-Alarcón, 2018 [22]	Cross-sectional	22	18	5 (22%)	79.6	60	99	79	80.2
Simpson et al., 2019 [23]	Prospective	732	218	514 (70%)	70.1	18	x	69.1	70.2
Hickson et al., 2014 [24]	Retrospective	160	85	75 (46%)	73	60	91		
Bainbridge et al., 2014 [25]	Cross-sectional	1636	541	1095 (33%)		70			
Gallagher, 2018 [26]	Cross-sectional	32	12	22 (64%)	71.6	47	80	71.5	76.7
Hong, 2014 [27]	Retrospective	1318	1237	81 (6%)	65.3				
Assi et al., 2021 [28]	Cross-sectional	5146	1587	3559 (69%)					
Garstecki et al., 1998 [29]	Cross-sectional	131	60	71 (54%)	74.5	65	90	75.35	73.7
Solheim et al., 2019 [30]	Prospective	181	153	28 (15%)	79.2	60			
Korkmaz et al., 2016 [31]	Retrospective	400	351	49 (12%)	63.67	39	89		
Aazh et al., 2015 [12]	Cross-sectional	1023	727	296 (28%)	74			75	73
Harumi et al., 2015 [32]	Cross-sectional	305	267	38 (12%)	69	21	101	71.33	69.71
Lee et al., 2015 [33]	Retrospective	119	81	38 (31%)	58	19	81	61.4	57.3
Oyarzún et al., 2017 [34]	Cross-sectional	78	54	24 (30%)	77.4	65			
Bulgurcu et al., 2019 [35]	Retrospective	191	60	131 (68%)	77.54	60			
Abdellaoui et al., 2013 [36]	Prospective	184	99	85 (46%)	74.2	55	92		
Kaplan-Neeman et al., 2012 [37]	Cross-sectional	177	146	31 (17%)	65.7	49		65.2	66.2
Maeda et al., 2016 [38]	Retrospective	157	91	66 (42%)	75.3	65		74.6	76.1
Maul et al., 2011 [10]	Cross-sectional	208	123	85 (40%)	74.6	65			

**Table 3:** Causes for use and rejection of hearing aids.

Author, Year	Causes for Use	Causes for Rejection	Causes for Use Legend	Causes for Rejection Legend
Humes et al., 2021 [20]	1, 2		1: a higher degree of hearing loss (in each article screened, they classify the degree of hearing differently) or bilateral hearing loss. 2: greater awareness of their condition. 3: greater satisfaction with the device, ease of use of the device, or having a simpler or more modern device. 4: higher economic status, higher income, higher level of education, having a university degree (each article examined determined in a different way the economic level qualified as high). 5: social issues (working age, daily activity, difficulties communicating in daily life, social support). 6: emotional causes, having a positive attitude. 7: greater experience using the device, using the device more hours per day. 8: being caucasian 9: recent diagnosis, recent audiometry evaluation, regular medical monitoring. 10: older age. 11: being male. 12: other comorbidities.	1: lack of awareness of their condition. 2: low or no perceived benefits from hearing aid use. 3: inability to understand others. 4: finding the device uncomfortable or difficult to use. 5: social stigma and other social causes. 6: lack of sufficient income. 7: lack of social or family support. 8: older age.
Fuentes-López, 2019 [21]	2, 3, 4	2, 3, 4		
Carrasco-Alarcón, 2018 [22]		2, 3, 4		
Simpson et al., 2019 [23]	1, 2, 4, 5, 6, 8			
Hickson et al., 2014 [24]	2, 3, 5, 6, 7	2, 3		
Bainbridge et al., 2014 [25]	1, 2, 4, 9			
Gallagher, 2018 [26]	1, 2, 5, 7, 10	2, 3, 4, 5		
Hong, 2014 [27]		2, 3, 4, 6		
Assi et al., 2021 [28]	3, 4, 5, 6, 8, 10, 11, 12			
Garstecki et al., 1998 [29]	1, 2	5, 7		
Solheim et al., 2019 [30]	2, 12	2, 4		
Korkmaz et al., 2016 [31]	1, 7			
Aazh et al., 2015 [12]	1, 10	3, 2		
Harumi et al., 2015 [32]	7, 4			
Lee et al., 2015 [33]	3, 5			
Oyarzún et al., 2017 [34]	1			
Bulgurcu et al., 2019 [35]		2, 3, 4, 6		
Abdellaoui et al., 2013 [36]	2, 3, 5	2, 6		
Kaplan-Neeman et al., 2012 [37]	1, 7, 10			
Maeda et al., 2016 [38]	6			
Maul et al., 2011 [10]	3			

## Data Availability

Unavailable data.
